# Itch-associated peptides: RNA-Seq and bioinformatic analysis of natriuretic precursor peptide B and gastrin releasing peptide in dorsal root and trigeminal ganglia, and the spinal cord

**DOI:** 10.1186/1744-8069-10-44

**Published:** 2014-08-14

**Authors:** Samridhi C Goswami, Danielle Thierry-Mieg, Jean Thierry-Mieg, Santosh Mishra, Mark A Hoon, Andrew J Mannes, Michael J Iadarola

**Affiliations:** 1 Department of Perioperative Medicine, Building 10, Room 2C401, MSC 1510, Clinical Center, NIH, 10 Center Drive, Bethesda, MD 20892, USA; 2 National Center for Biomedical Information, NIH, Bethesda, MD, USA; 3 Molecular Genetics Unit, Laboratory of Sensory Biology, National Institute of Dental and Craniofacial Research, NIH, Bethesda, MD, USA

**Keywords:** Pruritis, Bombesin, Ranatensin, GRP, GRPR, NMB, NMBR, Tac1, Nppa, Nppb, Nppc, Npr1, Npr2, Npr3, RNA-Seq, PolyA^+^ mRNA, RNA-Seq unified mapper, Peptidylglycine alpha-amidating monooxygenase, Immunocytochemistry, Cross-reaction

## Abstract

**Background:**

Three neuropeptides, gastrin releasing peptide (GRP), natriuritic precursor peptide B (NPPB), and neuromedin B (NMB) have been proposed to play roles in itch sensation. However, the tissues in which these peptides are expressed and their positions in the itch circuit has recently become the subject of debate. Here we used next-gen RNA-Seq to examine the expression of transcripts coding for GRP, NPPB, NMB, and other peptides in DRG, trigeminal ganglion, and the spinal cord as well as expression levels for their cognate receptors in these tissues.

**Results:**

RNA-Seq demonstrates that GRP is not transcribed in mouse, rat, or human sensory ganglia. NPPB, which activates natriuretic peptide receptor 1 (NPR1), is well expressed in mouse DRG and less so in rat and human, whereas NPPA, which also acts on the NPR1 receptor, is expressed in all three species. Analysis of transcripts expressed in the spinal cord of mouse, rat, and human reveals no expression of *Nppb*, but unambiguously detects expression of *Grp* and the GRP-receptor (*Grpr*). The transcripts coding for NMB and tachykinin peptides are among the most highly expressed in DRG. Bioinformatics comparisons using the sequence of the peptides used to produce GRP-antibodies with proteome databases revealed that the C-terminal primary sequence of NMB and Substance P can potentially account for results from previous studies which showed GRP-immunostaining in the DRG.

**Conclusions:**

RNA-Seq corroborates a primary itch afferent role for NPPB in mouse and potentially NPPB and NPPA in rats and humans, but does not support GRP as a primary itch neurotransmitter in mouse, rat, or humans. As such, our results are at odds with the initial proposal of Sun and Chen (2007) that GRP is expressed in DRG. By contrast, our data strongly support an itch pathway where the itch-inducing actions of GRP are exerted through its release from spinal cord neurons.

## Introduction

The concept of a specific circuit for the sensation of itch received a strong impetus from two papers in 2007 and 2009 [1,2]. These studies described how gastrin releasing peptide (GRP) can specifically cause scratching behavior which is lost upon genetic or pharmacological elimination of the GRP-receptor or GRP-receptor bearing second order neurons in the spinal cord. The behavior evoked by GRP was claimed to be due to the release of this peptide from primary afferents which in turn was proposed to cause the activation of GRP-receptor-bearing neurons in the spinal cord dorsal horn [1,2]. Recently, however the expression of GRP in DRG has been disputed [3,4] and a role for another neuropeptide, natriuretic precursor peptide B (NPPB), in provoking scratching behavior was delineated in mice [4]. Additionally, the capacity for a third neuropeptide, neuromedin B (NMB) to evoke scratching behaviors has been suggested [5].

In the present report, we address the question of expression of *Grp*, *Nppb*, and *Nmb* and their receptors in DRG and spinal cord through application of RNA-Seq. This method has several notable advantages over other techniques such as gene-array, RT-PCR and *in situ* hybridization (ISH). First, RNA-Seq uses next-generation DNA sequencing to determine every mRNA transcript in a particular sample. Since each sequenced cDNA fragment can be counted, the resulting data are quantitative. Second, because the association of transcript fragments to a particular gene is based on exact sequence alignment, rather than hybridization, gene identification is exquisitely precise. Third, the presence of rare transcripts can be captured by adjusting the depth of sequencing, thereby providing a rigorous measurement for all, or nearly all, expressed genes in a sample. These properties allowed us to accurately assess the expression level of *Nppb*, *Grp*, and *Nmb* and their cognate receptors.

For sensory ganglia, we examined expression in mouse and rat DRG, and mouse, rat and human trigeminal ganglia. We also determined expression of these genes in mouse, rat and human spinal cord. For mouse DRG, a genetic labeling strategy was used to isolate neurons of the transient receptor potential family V, member 1 (TRPV1) lineage and the non-TRPV1 lineage [6]. The latter DRG preparation consists of neurons remaining following ablation of neurons in the TRPV1-lineage. The neuropeptides GRP, NPPB and NMB co-localize with TRPV1-expressing cells or the TRPV1-lineage of nociceptive neurons [7]. Furthermore, it has been shown that the TRPV1-lineage neurons are absolutely required for pruritic responses [4]. These two preparations were extensively sequenced (~172 million paired end reads) to reveal transcripts expressed even at very low levels. Thus, the depth of sequencing combined with our genetic enrichment strategy enhances the chances of identifying rare transcripts in mouse DRG, including those for *Grp*. Importantly, for comparison we also provide data on a class of mouse sensory neurons not required for itch-behavior (the non-TRPV1 transcriptome). In all other cases, we sequenced the entire DRG or trigeminal ganglion to a lower extent (~40 million paired end reads) which is sufficient to detect low expressed genes.

Our results demonstrate that mouse, rat, and human sensory neurons express natriuretic peptides capable of activating NPR1, while a role for GRP as a primary afferent itch neurotransmitter is not supported.

## Methods

### Animal and human tissues

The mouse and rat PNS and CNS tissues analyzed in this study were obtained under protocols approved by the institutional Animal Care and Use Committees of the National Institute of Dental and Craniofacial Research and the Clinical Center, NIH. The human trigeminal ganglia and spinal trigeminal nucleus caudalis tissues were collected as part of the National Institute of Mental Health Brain Bank program.

### RNA sources

Mouse TRPV1 lineage and non-TRPV1 lineage DRG RNA samples were obtained from BAC-TRPV1 promoter-Cre mice as described (Mishra et al., 2011). Total RNA was extracted using the RNeasy Mini kit (Qiagen Inc., Valencia, CA, USA) and the RNA integrity was assessed by gel electrophoresis on an Agilent Bioanalyzer. Samples with a RNA integrity number (RIN) above 8.5 were sequenced from individual rat DRG (N = 9), rat DH (N = 3), rat TG (N = 3), mouse DRG lineage preparations (N = 2), mouse DH (N = 4), mouse TG (N = 3), human TG (N = 2) and human DH (N = 3). N represents individual samples; for the individual rat DRG samples, we pooled L4, L5 and for the individual mouse TG samples, both TGs were pooled. Values are reported as average ± SEM RPKM where three or more samples are available. Rat and mouse spinal cord were dissected from the dorsal half of the lumbar enlargement and human spinal cord RNA was prepared from the trigeminal nucleus caudalis at the level of the pyramidal decussation. Next-Gen Sequencing was performed on cDNA from poly A+ selected mRNA using the Illumina HiSeq2000 platform using version 3 chemistry according to standard protocols. For the TRPV1-lineage and non-TRPV1 lineage neuronal preparations, 166 and 178 million paired end reads of 101 bases were obtained, respectively; all other datasets were sequenced to ~40 million paired end reads of 101 bases. We also analyzed RNA-Seq data for rat DRG from in-silico data deposited by Hammer et al. (2010) in conjunction with their spinal nerve ligation study [8].

### RNA-Seq

Quality control was performed on all raw sequence data using FastQC (version 0.10.0). The minimum FastQC score for all the datasets was above or equal to 28 and therefore no further preprocessing was done. The sequences were aligned and mapped with RNA-Seq unified mapper (RUM v2.05) [9]. Gene annotations from Ref-seq, UCSC and ENSEMB were used within RUM. To account for variability in expression values between mapping algorithms, all the samples were remapped with another tool, MAGIC <ftp://ftp.ncbi.nlm.nih.gov/repository/acedb/Software/Magic>, which uses the AceDB for genome annotation <http://www.acedb.org/index.shtml>. Data values are presented as Reads Per Kilobase of exon model per Million mapped reads (RPKM), a normalization which takes into consideration the length of coding exons of genes and depth of sequencing [9].

### Bioinformatic search for GRP-like peptides

Considering that *Grp* is not expressed in DRG, we conducted a proteomic-bioinformatic search to formally address the question of: What peptides are similar enough to GRP to account for the immunoreactive material in DRG neurons observed by immunofluorescence [1]? We focused on peptides with C-termini similar to GRP because many studies use antisera generated to the carboxy-end of the 14 AA amphibian peptide bombesin (pGlu-Gln-Arg-Leu-Gly-Asn-Gln-Trp-Ala-Val-Gly-His-Leu-Met-NH2). C-terminal amidation is known to proceed via the enzyme peptidylglycine α-amidating monooxygenase (PAM), which cuts the precursor polypeptide such that the nitrogen atom from an adjacent glycine residue is transferred to the amidated amino acid [10,11]. In the precursor, the glycine is usually followed by paired basic residues typical of peptide-containing polyproteins. Specifically, based on these considerations we used the ProSite tool to search the SwissProt portion within the UniProtKB database for the following sequences GHLMG(KR)(KR), HLMG(KR)(KR), or XMG(KR)(KR). These search entries contain the cryptic peptide sequence motifs required for the C-terminal amidation resulting in a C-terminal methionine-amide.

## Results

### Dorsal root and trigeminal ganglia

#### *Neuropeptide expression in dorsal root/trigeminal ganglia*

Our RNA-Seq analysis employed both RUM and MAGIC which yielded similar results. For simplicity, data acquired by RUM are presented (Tables 1 and 2). Results obtained from analysis of DRG (mouse and rat), and TG (mouse, rat, and human) show that *Grp* is *not* expressed in primary sensory neurons (Table 1). We base this conclusion on the fact that no raw reads for *Grp* transcripts were detected in mouse or rat DRG preparations with RUM or MAGIC. We also analyzed a completely independent data by Hammer et al. (2010), which also showed no GRP expression in DRG (not shown). In the aggregate, we have analyzed 17 DRG or TG samples from three species, as well as the Hammer dataset. With this large number of samples, it is highly unlikely that the lack of observable *Grp* expression is due to sample under-representation or to species-specific differences in expression of *Grp*.

**Table 1:** Neuropeptide and receptor expression in dorsal root ganglia and trigeminal ganglia

	**Dorsal root ganglion**			**Trigeminal ganglion**		
**Gene symbol and gene name**	**Mouse TRPV1 lineage RPKM**	**Mouse non-TRPV1 lineage RPKM**	**Rat whole ganglion RPKM**	**Mouse whole ganglion RPKM**	**Rat whole ganglion RPKM**	**Human whole ganglion RPKM**
Grp, gastrin-releasing peptide	-	-	-	-	-	0.1
Grpr, gastrin releasing peptide receptor	0.2	0.2	-	0.3 ± 0	0.1 ± 0	0.1
Nmb, neuromedin B	170.3	73.7	32.6 ± 4.49	16.5 ± 0.9	13.0 ± 0.3	7.5
Nmbr, neuromedin B receptor	0.1	0.1	0.2 ± 0.02	0.1 ± 0	0.3 ± 0	0.2
Nppa, natriuretic peptide A; ANF	1.5	1.2	5.1 ± 0.73	1.0 ± 0.1	0.8 ± 0.1	1.8
Nppb, natriuretic peptide B; BNP	24.1	0.2	0.7 ± 0.14	2.0 ± 0.1	-	0.8
Nppc, natriuretic peptide C; CNP	-	0.1	-	0.1 ± 0	0.1 ± 0	0.4
Npr1, natriuretic peptide receptor 1; Npra	0.7	1.2	0.8 ± 0.06	1.2 ± 0	0.9 ± 0	0.9
Npr2, natriuretic peptide receptor 2; Nprb	38.1	30	31.7 ± 0.85	35.4 ± 0.4	36.7 ± 0.8	15.3
Npr3, natriuretic peptide receptor 3; Nprc	0.1	0.8	4.1 ± 0.44	2.6 ± 0.1	4.3 ± 0.5	0.3
Tac1, protachykinin-1	597.7	14.1	140.3 ± 10.92	59.8 ± 2.84	122.2 ± 4.9	44.1

The values in the table are average expression levels (and where applicable ± SEM) in RPKM of itch related neuropeptides and their receptors. RPKM is an acronym for Reads Per Kilobase of exon model per Million mapped reads, a normalization which takes into consideration the length of coding exons of genes and depth of sequencing [9]. The mouse DRG dataset consists of TRPV1 lineage and non-TRPV1 lineage RNA samples which were obtained from BAC-TRPV1 promoter-Cre mice as described (Mishra et al., 2011) [6]. Annotation builds mm10, rn4, and hg19 were used, respectively, for the mouse, rat and human genome mapping. Dashes (-) represent values of 20 reads or below; this number of reads is too inaccurate for further consideration.

**Table 2:** Neuropeptide and receptor expression in dorsal spinal cord

	**Dorsal spinal cord**		
**Gene symbol and gene name**	**Mouse RPKM**	**Rat RPKM**	**Human RPKM**
Grp, gastrin-releasing peptide	1.58 ± 0.71	2.09 ± 0.33	2.93 ± 0.39
Grpr, gastrin releasing peptide receptor	1.34 ± 0.15	0.94 ± 0.10	0.38 ± 0.03
Nmb, neuromedin B	1.28 ± 0.16	0.51 ± 0.08	2.70 ± 0.29
Nmbr, neuromedin B receptor	1.97 ± 0.11	2.02 ± 0.27	2.71 ± 0.72
Nppa, natriuretic peptide A; ANF	0.26 ± 0.03	1.21 ± 0.22	1.02 ± 0.07
Nppb, natriuretic peptide B; BNP	0.02 ± 0.01	0.01 ± 0.01	0.85 ± 0.21
Nppc, natriuretic peptide C; CNP	4.64 ± 0.66	3.61 ± 0.71	54.83 ± 9.74
Npr1, natriuretic peptide receptor 1; Npra	1.75 ± 0.17	5.94 ± 0.46	0.19 ± 0.05
Npr2, natriuretic peptide receptor 2; Nprb	12.46 ± 0.30	19.97 ± 4.90	5.79 ± 0.36
Npr3, natriuretic peptide receptor 3; Nprc	0.77 ± 0.08	0.81 ± 0.13	0.05 ± 0.01
Tac1, protachykinin-1	14.74 ± 1.55	16.14 ± 3.27	70.25 ± 7.60

Average expression levels (±SEM) in dorsal spinal cord of itch-related neuropeptides and their receptors for mouse, rat and human are shown. Detectable expression of *Grp*, *Grpr*, *Nmb*, and *Nmbr* is seen in all three species. *Tac1* is well expressed in spinal cord tissue [24], especially in the human. RPKM levels were determined by Rapid Unified Mapper (RUM) [9]. Annotation builds mm10, rn4, and hg19 were used, respectively, for the mouse, rat and human genome mapping. Dashes (-) represent values of 20 reads or below; this number of reads is too inaccurate for further consideration.

In contrast to GRP, we found that three other neuropeptides are highly expressed in mouse DRG and TG (Table 1). Specifically in TRPV1 lineage-neurons, the RPKM values for *Tac1* (coding for substance P), *Nmb*, and *Nppb* were 597.7, 170.3, and 24.1, respectively. In contrast, *Nppb* was virtually absent in non-TRPV1-neurons whereas *Tac1* and *Nmb* showed some overlap. NMB, Substance P and NPPB have been implicated as mediators of nociceptive processes [4,7,12]. The itch-specific neurotransmitter NPPB has two other paralogs NPPA and NPPC. *Nppa* is expressed at 5.1 RPKM in rat DRG, and approximately 1 RPKM in mouse and human sensory neurons. On the other hand, *Nppc* is expressed below 0.5 RPKM in mouse, rat, and human sensory ganglia.

#### *Neuropeptide receptors in dorsal root/trigeminal ganglia*

In mouse, rat and human DRG and TG, the receptors for GRP and NMB are expressed at very low levels (0.1-0.3 RPKM, Table 1). Generally, this level of expression is not considered to be functionally relevant. The NPR1 receptor, which binds natriuretic peptides NPPA and NPPB [13,14], is expressed at very low levels in sensory neurons (0.7-1.2 RPKM for mouse, rat and human DRG and TG). NPR3, the scavenger receptor for all the natriuretic peptides [14], shows a more variable distribution of expression, ranging between 0.1 and 4.3 RPKM in mouse, rat and human DRG and TG. In contrast, the receptor for NPPC, NPR2 [14], is highly expressed in all ganglia and species.

### Spinal cord

#### *Neuropeptide expression in spinal cord*

The fact that we did not detect *Grp* expression in DRG is not because RNA-Seq lacks the sensitivity to detect the transcript for *Grp* as we readily measured its expression in the dorsal spinal cord of mouse, rat and human (average of 2.2 RPKM). *Grp* is expressed at a similar level to *Nmb* in the spinal cord (1.59 RPKM average in the three species). In contrast, *Nppb* is not expressed or is only expressed at trace levels (~0.01 RPKM) in mouse and rat dorsal spinal cord and is low (~0.85 RPKM) in human dorsal spinal cord. The other two genes in the natriuretic peptide family, *Nppa* and *Nppc* are both expressed in dorsal spinal cord, with *Nppc* being expressed at a higher level, approximately 4 RPKM in mouse and rat and substantially greater in human (54.8 RPKM). A species difference is also seen for *Nppa*, with the lowest expression level (0.26 RPKM) in mouse and slightly higher expression (~1.1 RPKM) in rat and human. These data suggest that NPPA and NPPC may both participate in some aspects of sensory modulation.

#### *Neuropeptide receptor in spinal cord*

The receptor for GRP is expressed in dorsal spinal cord at approximately similar levels in mouse and rat (average of 1.1 RPKM). Lower levels are seen in human 0.38 RPKM. NMB receptors are expressed at a slightly higher level (average of 2.23 RPKM) compared to the GRP receptors, and levels are similar across mouse, rat and human. For the natriuretic peptide receptor, NPR2, mice, rats, and humans have RPKM values of 12.5, 20, and 5.8, respectively. Transcript levels of *Npr1* and *Npr3* are comparatively less in the spinal cord. *Npr1* is expressed at an average of 3.85 RPKM in mouse and rat and 0.19 RPKM in human and *Npr3* is expressed at an average of 0.79 RPKM in mouse and rat and at trace level (0.05 RPKM) in human. While the lumbar dorsal spinal cord was dissected in mouse and rat, we note that our human samples were collected from a more rostral level (spinal trigeminal nucleus at the decussation of the pyramidal tract) than in the rat or mouse. This difference in rostro-caudal level may account for some of the differences in transcript expression observed between species.

#### *Bioinformatic identification of GRP-like peptides*

**Figure 1:**
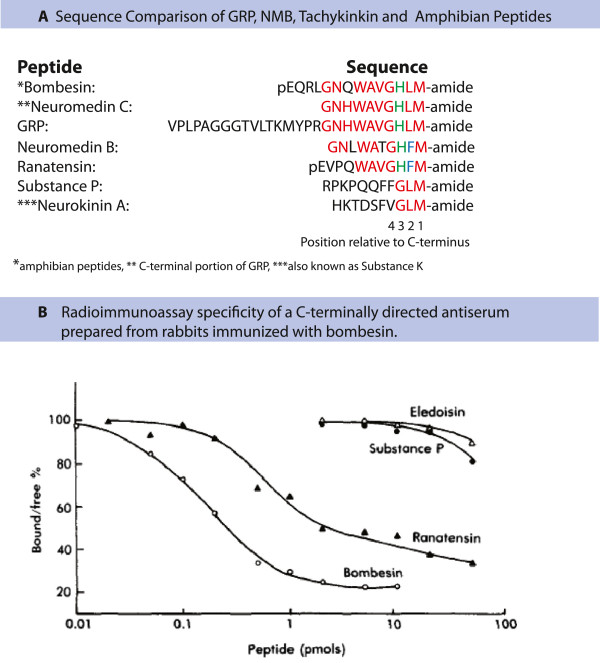
**Evaluation of sequences and cross reaction of GRP/bombesin related neuropeptides. A**: Amino acid sequence comparison of GRP, NMB, Tachykinin and amphibian peptide orthologs. The table depicts amino acid sequences obtained from human and amphibian sources [http://www.ncbi.nlm.nih.gov/protein]. There is conservation of sequence across human, mouse and rat species for all peptides other than GRP. Mouse and rat GRP share 100% sequence identity which is: VSTGAGGGTVLAKMYPRGSHWAVGHLM (differences from the human sequence are highlighted in red). **B**: The competitive binding figure is reproduced with permission from Panula et al. (1982) [27]. It examines the cross-reaction of a C-terminally directed anti-bombesin antiserum with bombesin, ranatensin, substance P and eledoisin (an amphibian peptide similar to substance P). Ranatensin shares C-terminal sequence similarity with Neuromedin B and fully displaces the iodinated bombesin tracer. Substance P is just beginning to displace the tracer at the concentrations used.

Previously, it has been suggested from immunohistogical studies that *Grp* is expressed in sensory ganglia [1,2]. We asked whether the difference between these immunohistochemical observations and our quantitative RNA-Seq analysis could be attributed to cross-reaction of antibodies with other neuropeptides. A bioinformatics approach was devised to search for possible peptide sequences that may cross-react with C-terminally directed bombesin/GRP-antibodies and searched protein databases for two peptide sequences, GHLMG(KR)(KR) and HLMG(KR)(KR) because they contain the cryptic C-terminal amidation signal [10,11]. This stringent search yielded matches only to bombesin and GRP (Figure 1A). When we reduced the stringency to XMG(KR)(KR) we obtained matches to ~300 proteins. Searching this data set for neuropeptides that are expressed in DRG and TG, we found matches to two neuropeptides, NMB and Substance P as well as matches to peptide analogs isolated from frog skin (Figure 1A) [15]. These data raise the possibility that NMB and/or substance P may cross react with C-terminally directed anti-bombesin/Grp antibodies. This possibility is strongly supported by the very high expression of these two peptides in DRG and had been proposed in an earlier study [3]. In fact, our RNA-Seq data disclose enormously high level of *Nmb* and *Tac1* (Substance P-containing) transcripts (170 and 597 RPKM, respectively; Table 1) which could act as rich targets for antibody cross-reactivity.

## Discussion

The understanding of the neurobiology of itch has progressed considerably over the last 5 years with the identification of a number of neuropeptides critical for itch-responses. Gene knockout, cell deletion, pharmacological, and immunohistochemical or *in situ* hybridization data have been assembled to delineate the functions and neuronal localization of three neurotransmitters, GRP, NPPB, and NMB, in the primary afferent-spinal cord circuit. Several recent studies have emphasized a primary afferent role for NPPB [4] and NMB [3] in itch and deemphasized a primary afferent role for GRP [3,4]. More recently still, a primary afferent role for GRP has been re-emphasized [16]. To address this controversy directly, we analyzed expression levels of these neuropeptides in mouse, rat and human DRG/TG and dorsal spinal cord using RNA-Seq. The RNA-Seq methodology provides unequivocal gene identification and exact quantification of gene expression, even when a gene is expressed at very low levels. We also used these data to examine expression levels of all the relevant receptor paralogs that might be expressed in mouse, rat and human DRG/TG and dorsal spinal cord to obtain a complete expression profile within the neuronal circuit and across species.

Deep sequencing, in combination with a FACS strategy for enrichment of the TRPV1 neuronal lineage population, a subset of sensory neurons which are known to be required for pruritic responses [4], did not detect *Grp* transcripts. Furthermore, analyses of multiple whole rat DRG/TG and human TG samples did not identify *Grp* transcripts. Thus, our RNA-Seq analyses indicate an absence of *Grp* expression in DRG and TG, consistent with several previous observations using different techniques [4,17].

### Transcription-based results

#### *DRG/TG*

The present quantitative RNA-Seq data indicates no expression of GRP in mouse, rat or human DRG/TG. An earlier *in situ* hybridization investigation also detected abundant expression of *Nmb* but no expression of *Grp* in rat DRG [17]. More recently two additional studies, also using ISH, failed to detect *Grp* in mouse DRG [3,4]. Furthermore, RT-PCR of DRG cDNA amplified either no or, only trace, amounts of *Grp*[3,4]. Taken together and viewed together with the perspective of our RNA-Seq measurements, these data strongly suggest that any equivocal observations of *Grp* expression in DRG are likely false positive results. While we cannot be certain of the reasons for false positives, possible explanations might include trace contamination of PCR primer sets or template preparations [18] or over-development of histochemical reactions.

It is important to note that expression of transcripts may differ in preparations that have been manipulated such as after ganglionic dissociation for primary cultures or transgenic manipulations. For example, primary mouse DRG cultures have been reported to express Npr3 whereas our RNA-Seq results from freshly dissected DRG tissue show quite low expression [19]. Over expression of *Braf* has also been reported to induce *Grp* expression in DRG [16]. Manipulations of afferent transmission by tissue inflammation or direct nerve injury may also cause altered expression, however, preliminary results obtained in rat from our RNA-Seq data after axotomy or hind paw inflammation do not show an induction of *Grp* or Grp-receptor in DRG.

The quantitative data for transcripts coding for GRP, NPPB, and NMB are presented in Table 1. Examination of these data across species indicates that *Nppb*, while well expressed in mouse, is expressed at a relatively low level in rat and human DRG/TG. In its place, the close family member, *Nppa* is expressed. Therefore, it is tempting to speculate that the rat and human itch circuit may utilize the same natriuretic-receptor (NPR1) as is used by mice, but instead of using NPPB, may use NPPA as a primary afferent itch co-transmitter. *Nmb* is expressed at high levels in mouse, rat and human DRG/TG suggesting an important conserved nociceptive function for this neuropeptide.

#### *Spinal cord*

The RNA-Seq results obtained from dorsal spinal cord illustrate the plethora of receptors and neuropeptides that are present in the neurons that integrate somatosensory signals. In the spinal cord, we detected expression of *Grp*, Grp-receptor (*Grpr*), *Nmb*, Nmb-receptor (*Nmbr*), *Nppa* and *Nppc* and the natriuretic receptors 1, 2 and 3. By contrast, there was almost no expression of *Nppb*. The present data are consistent with behavioral results obtained by intrathecal injections of various itch-inducing peptides, but do not necessarily clarify issues of circuitry or co-localization [19-22]. While we demonstrate the presence of the relevant molecular substrates, it is beyond the scope of the present study to critically evaluate behavioral results from earlier studies [1,2,4-7,16,19,23]. However, our results are consistent with most available neuroanatomical studies examining these targets in DH [4,17,24].

The RNA-Seq results also clarify the expression of certain receptors whose presence in spinal cord might have been difficult to interpret based on in situ hybridization or RT-PCR methods. The detection of *Nmb* and Nmb receptor (*Nmbr*) expression at levels approximately equal to *Grp* and Grp receptor (*Grpr*) expression suggests that the inability to detect *Nmb* and *Nmbr* in the dorsal spinal cord might be due to technical reasons [17]. Thus, the expression of multiple itch-related neuropeptides and their cognate receptors in the spinal cord along with evidence of NMB binding to the GRP receptor and GRP binding to the NMB receptor, albeit to a low affinity [22,25], allows for many plausible itch receptor-ligand interactions in this CNS region, and emphasizes issues related to dose–response in experimental design. These quantitative considerations can also be informative for further neurophysiological characterizations of spinal cord second order neurons [26].

### Neuropeptides and immunoreactivity

#### *DRG/TG*

Based on our RNA-Seq results we examined what might account for previous immunostaining reports that describe GRP-containing neurons in DRG [1,2]. Searches for sequences with similar primary structure to the antigen used to produce anti-bombesin/GRP antibodies revealed two candidates that can cross-react, NMB and Substance P. Both of these peptides are present in very high quantities in DRG neurons. Previously the immunocross-reactivity of various peptides has been examined for their potencies to displace [^125^I] bombesin from a C-terminally directed antibody (See Figure 1B; reproduced with permission from Panula et al., 1982) [27]. This study shows that, although the binding of ranatensin/NMB and substance P is far lower than that for Bombesin/GRP (between 10- to 200-fold, respectively), with sufficient peptide, full displacement of ^125^I-bombesin tracer can be obtained, especially for NMB, and with extrapolation, also for substance P [27]. The antibody used by Sun et al., and Chen et al., was a C-terminally directed anti-bombesin antibody, therefore the most plausible explanation for the discrete immunostaining of DRG neuronal cell bodies in these studies appears likely to be cross-reaction with NMB and/or substance P.

### Concluding remarks

Our data indicate that *Grp* is *not* expressed in DRG or TG. A reasonable explanation for immunofluorescent staining reported for GRP in DRG/TG could be that anti-GRP antibodies cross-react with Nmb and/or substance P due to the high levels of expression of the latter two neuropeptides and to the close primary structure of their amidated C-termini. As a consequence of this misidentification, several types of studies, such as innervation of the dermis and epidermis [28] and the effects of bombesin on wound healing, may need reinterpretation [29,30]. RNA-Seq provides information on gene expression only, and the fine details of protein turnover, subcellular compartmentalization, and/or localized enrichment are additional important considerations for integrative neurobiological research. Nonetheless, RNA-Seq provides a powerful new method for guiding research and a useful baseline to begin functional experimentation.

## Competing interest

There are no actual or potential conflicts of interest in relation to this article.

## Authors’ contributions

SCG performed bioinformatic components and manuscript preparation, DTM and JTM performed bioinformatic components and manuscript preparation, SM and MAH performed mouse genetic manipulations and manuscript preparation, AJM provided project oversight and manuscript preparation, MJI performed bioinformatic evaluations, neuropeptide expertise and manuscript preparation. All authors read and approved the final manuscript.
